# Cannabis use and other predictors of the onset of daily cigarette use in young men: what matters most? Results from a longitudinal study

**DOI:** 10.1186/s12889-015-2194-3

**Published:** 2015-09-02

**Authors:** Julia Becker, Michael P. Schaub, Gerhard Gmel, Severin Haug

**Affiliations:** Swiss Research Institute for Public Health and Addiction ISGF, University of Zurich, Konradstrasse 32, CH-8031 Zurich, Switzerland; Alcohol Treatment Centre, Lausanne University Hospital CHUV, Rue du Bugnon 21, CH-1011 Lausanne, Switzerland; Addiction Switzerland, Av. Louis-Ruchonnet 14, CH-1003 Lausanne, Switzerland; Centre for Addiction and Mental Health, Toronto, Ontario Canada; University of the West of England, Bristol, UK

## Abstract

**Background:**

According to the gateway hypothesis, tobacco use is a gateway of cannabis use. However, there is increasing evidence that cannabis use also predicts the progression of tobacco use (reverse gateway hypothesis). Unfortunately, the importance of cannabis use compared to other predictors of tobacco use is less clear. The aim of this study was to examine which variables, in addition to cannabis use, best predict the onset of daily cigarette smoking in young men.

**Methods:**

A total of 5,590 young Swiss men (mean age = 19.4 years, SD = 1.2) provided data on their substance use, socio-demographic background, religion, health, social context, and personality at baseline and after 18 months. We modelled the predictors of progression to daily cigarette smoking using logistic regression analyses (*n* = 4,230).

**Results:**

In the multivariate overall model, use of cannabis remained among the strongest predictors for the onset of daily cigarette use. Daily cigarette use was also predicted by a lifetime use of at least 50 cigarettes, occasional cigarette use, educational level, religious affiliation, parental situation, peers with psychiatric problems, and sociability.

**Conclusions:**

Our results highlight the relevance of cannabis use compared to other potential predictors of the progression of tobacco use and thereby support the reverse gateway hypothesis.

## Background

Many variables could be important for the progression of tobacco use. The identification of the relevant ones that best predict the progression of tobacco use is highly important because tobacco use is, by far, more widespread than cannabis and other illicit drug use and accounts for a significantly greater global burden of disease [1]. Identifying which young adults show a higher risk of transition to more involved stages of tobacco use would be helpful for indicative prevention efforts in this age group.

One of the discussed predictors of tobacco use is cannabis use. Tobacco use can act as a gateway to cannabis use [2], but the reverse has also been observed, i.e., cannabis use acting as a gateway to the initiation of tobacco use [3, 4]. Furthermore, the probability of progressing from occasional to regular tobacco smoking and nicotine dependence is higher in smokers who also use cannabis [4–6]. However, the underlying mechanisms connecting tobacco and cannabis use are less clear but are assumed to go beyond the mechanisms underlying the co-use of substances such as tobacco and alcohol in general [7]. Moreover, the relative importance of the mechanisms that contribute to the co-use of tobacco and cannabis may vary across development [8] and stages of use [9].

The way of substance administration is probably among the most important connecting mechanisms of tobacco and cannabis use. In the qualitative study by Amos and colleagues [10], many participants reported that smoking joints, i.e., co-administration of cannabis and tobacco, served as a gateway to smoking cigarettes. A study by Agrawal and Lynskey [11] underlined the importance of the way of administration in linking cannabis and tobacco use. In this study, smoking tobacco was significantly associated with cannabis use and dependence whereas the use of smokeless tobacco was not. Because of the shared route of administration, tobacco and cannabis smoking may serve as behavioural cues for one another and therefore reinforce one another [7]. Moreover, the cross-drug reinforcement of tobacco and cannabis use also occurs on a pharmacological level. Tobacco and cannabis affect the same neural pathways, with some systems being mutually enhanced by the two substances and others having contrasting effects [12]; additionally, nicotine may prolong and enhance the subjective effects of cannabis [3, 13]. The co-administration of cannabis and tobacco is the most widespread way of cannabis administration in many countries, such as Australia [14] and Switzerland, where 97.3 % of young cannabis using men reported mulling, i.e., smoking cannabis as joints mixed with tobacco [15]. In the United States, cannabis is often wrapped in a tobacco leaf and smoked as “blunts” [16] and the majority of cannabis users additionally smoke cigarettes [17]. It is therefore crucial to evaluate the importance of cannabis use in predicting the initiation of cigarette smoking or progression from occasional to regular cigarette smoking.

Apart from the way of administration, the common liability model can also explain the strong association between tobacco and cannabis use. This model assumes that a common liability to using both licit and illicit drugs puts an individual at risk for using both legal and illegal substances, such as tobacco and cannabis. This liability may include a genetic and individual vulnerability, such as proneness to deviant personality and familial liability to addiction [18]. Peer influences in adolescence appear to be one factor that influences individual vulnerability. By analysing the origins of the correlation between tobacco, alcohol, and cannabis use among adolescents, an Australian study found an individual’s vulnerability to substance use to be an explaining factor [19]. Vulnerability, in turn, was predicted by the extent to which the individual was affiliated with delinquent and substance using peers.

Other potential predictors that could influence tobacco and/or cannabis use are religiosity and context variables such as socio-economic status or changes in social environments. Religious and pro-social activities are negatively associated with late-onset cannabis use [11] whereas substantial gains or losses in religiosity from childhood to adulthood are positively associated with substance use and misuse in the general U.S. population [20]. A recent longitudinal study suggested that individuals who experienced a declining socio-economic position from childhood to adulthood may be twice as likely to use tobacco and cannabis compared to individuals with a stable trajectory [21].

The aim of this study was to explore how the onset of daily cigarette smoking can be best predicted from a comprehensive set of variables, including cannabis use.

## Methods

### Study design and procedure

The present data are part of the Cohort Study on Substance Use Risk Factors (C-SURF), a longitudinal study designed to assess substance use patterns and their related consequences in young Swiss men. Enrolment in the study occurred between 2010 and 2011 in three Swiss army recruitment centres, which cover 21 of the 26 Swiss cantons. (A canton is a type of administrative division of a country and the Swiss cantons are semi-sovereign states.) Switzerland has a mandatory army recruitment process: virtually all young men are contacted at approximately 19 years of age for determination of their eligibility for military or civil service. Thus, not only individuals who were finally selected to serve in the army were enrolled in the study, but a virtually complete census of the Swiss male population in this age group was eligible. The participants filled in the questionnaire online or via mail and were rewarded with a voucher of 30 Swiss Francs (CHF).

The follow-up assessment was conducted approximately 15 months after the baseline measurement, and the participants were reimbursed with a similar voucher. The participants who filled in both questionnaires received an additional voucher (30 CHF). Between the assessments, the participants were invited twice to update their contact details online. Each of these two updates was rewarded with a voucher (5 CHF) once the second questionnaire was completed.

The Ethics Committee for Clinical Research of Lausanne University Medical School approved the study (Protocol No. 15/07).

### Participants

A total of 15,074 young men visited the recruitment centres. Among them, 1,829 (12.1 %) did not meet the research staff because they were sick (but not chronically ill), were randomly selected to participate in another study [22], or were not informed about the study by the military staff. These non-inclusions were random and should not have influenced the findings. More information about sampling and non-response can be found in Studer et al. [23]. Of the 13,245 conscripts informed about the study, 7,563 (57.1 %) provided consent for participation, and 5,990 of those (79.2 %) completed the baseline questionnaire. The follow-up questionnaire was completed by 5,223 participants (87.2 %).

For the model of progression from no or occasional cigarette use at baseline to daily cigarette use at follow-up, we excluded 1,275 of the 5,990 individuals (21.3 %) because of daily cigarette use already at baseline and an additional 485 individuals (8.1 %) because of missing data, resulting in a final sample of 4,230 individuals.

### Measures

#### Outcome variable: onset of daily cigarette use

The participants indicating cigarette use during the previous 12 months were asked how often they usually smoke cigarettes. The possible answers (“every day”, “5–6 days per week”, “3–4 days per week”, “1–2 days per week”, “2–3 days per month”, and “once per month or less”) were dichotomised (daily vs. non-daily use). For the analysis of the onset of daily cigarette smoking, the sample included all the participants who used cigarettes less than daily or not at all at baseline. Among these participants, reporting daily cigarette smoking at follow-up was classified as the onset of cigarette use.

#### Predictor variables

All the predictor variables were measured at baseline.

##### Socio-demographics

The socio-demographic predictors included age in years and the highest completed level of education divided into two categories: a lower educational level (compulsory education or vocational school training) and a higher educational level (upper secondary education, college and university degrees). Additional socio-demographic predictors included the housing situation (living alone, living with a parent or parents, living with a partner, living with friends or in an institution), the means of subsistence (own person, own person and other persons or institutions, other persons or institutions), living in a partnership (yes/no), and the number of siblings.

##### Religion and religiosity

Religious denomination was assessed by the question “What is your religion (even if you do not practice or believe in God)?” with nine response categories, which we merged into four categories: Christian religion, Muslim religion, other religion, and no religion. To measure religiosity, we used the first question of the Religious Background and Behaviour Questionnaire (RBB) [24] with the response categories (1) “I believe in God and practice religion”, (2) “I believe in God but do not practice religion”, (3) “I do not know what to believe about God”, (4) “I believe we cannot really know about God” (agnostic), or (5) “I do not believe in God” (atheist).

##### Health and health behaviour

Physical and mental health were measured by the Physical Component Summary and the Mental Component Summary of the 12-Item Short-Form Health Survey (SF-12) [25], the Major Depression Inventory (MDI) [26], and the International Physical Activity Questionnaire (IPAQ) [27]. In a study using data from 9 different countries, correlations of both the Mental and the Physical Component Summary measures of the SF-12 and the SF-36 were between .94 and .97 [28]. Various studies have shown that the SF-36 is a valid and reliable measure of population health [28, 29]. A study of the psychometric properties of the MDI indicated adequate internal and external validity (high correlation of 0.86 with the Hamilton Depression Scale) [26].

##### Social context

The parental situation was assessed by a question derived from the Alcohol Use Disorder and Associated Disabilities Interview Schedule-IV (AUDADIS-IV) [30]. The parents’ educational level was assessed and categorised analogous to the educational level of the study participant (see above). The financial situation of the family was measured with a question from the European School Survey Project on Alcohol and Other Drugs (ESPAD) [31]; this question uses a 7-point scale (“very much below average”—“very much above average”) to assess how well off the individual’s family is compared to other Swiss families.

Parenting was assessed by three variables used in the ESPAD. Two questions (“Before you were 18 years old, how satisfied were you usually with your relationship with (a) your mother and (b) your father?”) measured the participants’ retrospective satisfaction with the relationship with their parents on scales ranging from 1 (“very satisfied”) to 5 (“not satisfied at all”). The scores were dichotomised at the median of the mean of the items assessing maternal and paternal relationships. Two questions with response scales ranging from 1 (“almost always”) to 5 (“almost never”) were used to derive parental regulation at the age of 15 years: “My parents set definite rules about what I was allowed to do (a) at home and (b) outside the home”. The scores were averaged and dichotomised at the median. The retrospective assessment of parental knowledge of peers and the whereabouts at age 15 were derived by averaging and dichotomising the scores obtained from the responses to two five-point items: “My parents knew (a) whom I was with and (b) where I was in the evenings.” Parental rule setting and knowledge of peers and the whereabouts was asked at around age 15 because this is the time when peer influences become stronger, and particularly meeting with friends without the participation of parent increases [32]. For example, the Study on Health Behaviour in School-Aged Children (HBSC) across 41 European countries showed that peer influences, such as being four or more days per week out with friends, increase strongly between the ages of 11 and 15 [33].

The lifetime prevalence of psychiatric, alcohol or drug problems in the parents was assessed separately for both parents. The participants indicated whether a significant problem had ever been present in one or several domains (i.e., psychiatric problem, alcohol problem, or drug problem). A similar question addressed previous significant alcohol, drug, or psychiatric problems in peers. Peer pressure was assessed by a shortened version of the Peer Pressure Inventory (PPI) [34].

##### Substance use

Lifetime use of alcohol was assessed by the question “Did you have at least 12 alcoholic standard drinks in your entire life?” Examples for alcoholic standard drinks were pictured. Furthermore, the age of the first use of at least one standard alcoholic drink, the 12-month prevalence, and at-risk drinking were assessed. The possible answers (“every day”, “5–6 days per week”, “3–4 days per week”, “1–2 days per week”, “2–3 days per month”, “once per month or less”) were dichotomised (daily vs. non-daily use). Participants who indicated ‘yes’ were classified by the Alcohol Use Disorders Identification Test (AUDIT-C) [35] as not at risk (score < 4) or at risk drinkers (score ≥ 4) [36]; participants who indicated ‘no’ were classified into the category ‘no alcohol use in the previous 12 months’. In studies, which compared the AUDIT-C to other, more comprehensive screening instruments for alcohol use disorders, the AUDIT-C showed good sensitivity, specificity and positive predictive validity [36, 37].

To assess the lifetime use of cigarettes, participants indicated whether they consumed at least 50 cigarettes in their life. Furthermore, the age of first cigarette smoking and the 12-month prevalence of cigarette smoking were assessed (see above). Additionally, the 12-month prevalence for the use of tobacco products other than cigarettes (i.e., water pipes (shisha, smoked only with tobacco), snus, snuff, chewing tobacco, cigars/cigarillos, tobacco pipes) was measured.

The lifetime use of cannabis was assessed by asking “Have you ever consumed cannabis (grass, hashish, marihuana), more than just to try?” Subsequent questions measured the age of first cannabis use and problematic cannabis use, which was assessed with the Cannabis Use Disorders Identification Test (CUDIT) [38]. Although the internal consistency of the CUDIT seems appropriate (.72–.78), the predictive power of the instrument, tested in different studies, is mixed [39]. A cut-off value of 8 was used to discriminate problematic from non-problematic cannabis use.

The lifetime use of illicit drugs other than cannabis at baseline was assessed by a series of questions measuring the frequency of use of 15 illicit drugs within the course of the individual’s life (e.g., hallucinogens, speed, amphetamines, crystal meth, poppers, ecstasy, cocaine/crack/freebase, and heroin). The lifetime use of illicit drugs was defined as having used at least one of these substances at least once.

##### Personality

Screening for adult attention deficit syndrome was performed with the Attention Deficit Syndrome Self Report Scale (ASRS-v1.1) [40]. Sensation seeking was measured by the Brief Sensation Seeking Scale (BSSS-8) [41]. In two studies, the BSSS-8 showed good internal consistencies (α = .76 and α = .74) and was predictive of other risk and protective factors [42]. Aggression/hostility, sociability and neuroticism/anxiety were assessed by the corresponding subscales of the Zuckerman-Kuhlman Personality Scale (ZKPQ-50-cc) [43]. In a validation sturdy, this instrument showed good psychometric and structural properties in four different languages with alpha coefficients above .70 [44]. Peer pressure was assessed by a shortened version of the Peer Pressure Inventory (PPI), which showed acceptable test-retest and inter-rater reliability in a study examining the perception of peer pressure [34]. The presence of an anti-social personality disorder (ASPD) was assessed by questions of the Mini International Neuropsychiatric Interview [45]. It involves two sections with six childhood criteria. If two of these criteria were positive, then the subjects were asked about six behaviours since age 15. Three affirmative answers qualified for ASPD.

### Analyses

Starting with separate logistic regression analyses (subsequently termed ‘univariate analyses’), we evaluated the potential of each baseline variable to predict the onset of daily cigarette use. To reduce multicollinearity within the final multivariate model, we developed separate multivariate prediction models for each of the following categories of predictor variables: (1) socio-demographics, (2) religion and spirituality, (3) health and health behaviour, (4) social context, (5) substance use, and (6) personality. Variable selection comprised the following steps: (1) Significant predictors from the univariate analyses were entered into the separate models. (2) Variables that were not significant were removed manually one by one; variables with the highest p-values were removed first (backward selection). (3) To account for suppressor effects, the resulting models were verified by tentatively adding the excluded variables separately. Only significant variables were retained in the category-specific multivariate models (forward selection). Based on the results of these models, we developed one final model for the onset of daily cigarette use. Variable selection was conducted in an analogous way as described above, with the exception of including all significant predictors from the category-specific models at step (1). Nagelkerke’s *R*^*2*^ was calculated as a goodness-of-fit measure for all multivariate models. All the analyses were performed using SPSS version 20 [46], and *p* < 0.05 was set as the significance level.

## Results

### Sample characteristics

The baseline characteristics of the 4,230 participants included in the analysis of the onset of daily cigarette smoking are displayed in Table 1. At baseline, 2,824 (66.8 %) participants reported no cigarette use during the preceding 12 months, whereas 1,406 (33.2 %) participants had smoked cigarettes occasionally. Among them, 216 (5.1 %) participants smoked five or six days per week, 139 (3.3 %) participants smoked three or four days per week, and 202 (4.8 %) participants smoked two or three days per month. Furthermore, 240 (5.7 %) participants had smoked cigarettes two or three days per month, and 609 (14.4 %) participants smoked monthly or less often.

**Table 1:** Baseline characteristics for individuals with and without the onset of cigarette use and univariate associations with the onset of daily cigarette use

Variable categories and variables	No onset *n* = 3961		Onset *n* = 269		*OR*	*(95 % CI)*	*P*
*Socio-demographics*							
Age in years, *M (SD)* ^a^	19.4	(1.2)	19.4	(1.2)	0.99	(0.89–1.10)	.827
Lower educational level (Ref) ^b^	2,758	(70.9 %)	220	(82.7 %)			
Higher educational level	1,132	(29.1 %)	46	(17.3 %)	0.51	(0.37–0.71)	<.001
Living with parent or parents (Ref) ^c^	3,598	(91.5 %)	232	(86.6 %)			
Living alone	98	(2.5 %)	10	(3.7 %)	1.58	(0.82–3.07)	.175
Living with partner	87	(2.2 %)	9	(3.4 %)	1.60	(0.80–3.23)	.185
Living with friends or in institution	151	(3.8 %)	17	(6.3 %)	1.75	(1.04–2.93)	.035
Means of subsistence: own person (Ref) ^d^	776	(19.7 %)	58	(21.7 %)			
Own person and others persons or institutions	1,643	(41.6 %)	129	(48.3 %)	1.06	(0.77–1.46)	.739
Other persons or institutions	1,522	(38.7 %)	80	(30.0 %)	0.70	(0.50–0.997)	.048
Not living in a partnership (Ref) ^e^	3,767	(95.8 %)	251	(94.0 %)			
Living in a partnership	166	(4.2 %)	16	(6.0 %)	1.45	(0.85–2.45)	.171
Having no siblings (Ref) ^f^	243	(6.3 %)	20	(7.7 %)			
One or two siblings	2,938	(76.6 %)	195	(75.3 %)	0.81	(0.50–1.30)	.378
Three or more siblings	656	(17.1 %)	44	(17.0 %)	0.82	(0.47–1.14)	.465
*Religion and spirituality*							
Christian religion (Ref) ^g^	2,949	(75.5 %)	176	(66.9 %)			
Muslim religion	142	(3.6 %)	10	(3.8 %)	1.18	(0.61–2.28)	.623
Other religion	87	(2.2 %)	6	(2.3 %)	1.16	(0.50–2.68)	.736
No religion	727	(18.6 %)	71	(27.0 %)	1.64	(1.23–2.18)	.001
Atheist (Ref) ^h^	1,025	(26.3 %)	86	(32.6 %)			
Agnostic	670	(17.2 %)	44	(16.7 %)	0.78	(0.54–1.14)	.202
Unsure what to think about god	491	(12.6 %)	37	(14.0 %)	0.90	(0.60–1.34)	.599
Believe in god but not practicing	1,201	(30.8 %)	77	(29.2 %)	0.76	(0.56–1.05)	.098
Believe in god and practicing	516	(13.2 %)	20	(7.6 %)	0.46	(0.28–0.76)	.002
*Health and health behaviour*							
Physical health (SF-12, scale 0–100), *M (SD)* ^i^	55.2	(5.0)	54.8	(5.2)	0.98	(0.96–1.01)	.133
Mental health (SF-12, scale 0–100), *M (SD)* ^j^	49.9	(8.4)	49.2	(9.0)	0.99	(0.98–1.01)	.220
Depression (MDI, scale 0–50), *M (SD)* ^k^	6.6	(6.7)	8.0	(8.0)	1.03	(1.01–1.04)	.001
Low physical activity (IPAQ) (Ref) ^l^	355	(9.6 %)	19	(7.8 %)			
Moderate physical activity	953	(25.9 %)	60	(24.7 %)	1.18	(0.69–2.00)	.548
High physical activity	2,371	(64.4 %)	164	(67.5 %)	1.29	(0.79–2.11)	.303
*Social context*							
Grew up with both parents (Ref) ^m^	3,181	(81.2 %)	185	(70.1 %)			
…with parent and step-parent	185	(4.7 %)	29	(11.0 %)	2.70	(1.77–4.10)	<.001
…with one parent	500	(12.8 %)	47	(17.8 %)	1.62	(1.16–2.26)	.005
…with adoptive or foster parents or in institution	51	(1.3 %)	3	(1.1 %)	1.01	(0.31–3.27)	.985
No parental divorce before the age of 18 (Ref) ^n^	3,066	(78.4 %)	183	(69.6 %)			
Parental divorce before the age of 18	846	(21.6 %)	80	(30.4 %)	1.58	(1.21–2.08)	.001
Lower educational level of the father (Ref) ^o^	1,998	(51.2 %)	133	(50.6 %)			
Higher educational level of the father	1,904	(48.8 %)	130	(49.4 %)	1.03	(0.80–1.32)	.842
Lower educational level of the mother (Ref) ^p^	2,241	(57.6 %)	157	(59.5 %)			
Higher educational level of the mother	1,653	(42.4 %)	107	(40.5 %)	0.92	(0.71–1.19)	.541
Financial situation of family (scale 1–7), *M (SD)* ^q^	3.56	(1.0)	3.6	(1.0)	1.04	(0.92–1.19)	.504
Good relationship with parents before age 18, (Ref) ^r^	3,217	(81.4 %)	199	(74.3 %)			
Bad relationship with parents before age of 18	733	(81.4 %)	69	(74.3 %)	1.52	(1.14–2.03)	.004
Lower parental rule setting at age 15 (Ref) ^s^	1,550	(39.3 %)	121	(45.3 %)			
Higher parental rule setting at age 15	2,398	(60.7 %)	145	(54.7 %)	0.78	(0.61–1.001)	.051
Lower parental monitoring at age 15 (Ref) ^t^	902	(22.8 %)	74	(27.8 %)			
Higher parental monitoring at age 15	3,046	(77.2 %)	192	(72.2 %)	0.77	(0.58–1.02)	.063
No psychiatric problem in the father (Ref) ^u^	3,697	(93.9 %)	246	(91.4 %)			
Psychiatric problem in the father	241	(6.1 %)	23	(8.6 %)	1.43	(0.92–2.24)	.114
No psychiatric problem in the mother (Ref) ^v^	3,783	(96.1 %)	252	(93.7 %)			
Psychiatric problem in the mother	154	(3.9 %)	17	(6.3 %)	1.66	(0.99–2.78)	.055
No psychiatric problem in peers (Ref) ^w^	2,398	(61.4 %)	116	(43.8 %)			
Psychiatric problem in peers	1,507	(38.6 %)	149	(56.2 %)	2.04	(1.59–2.63)	<.001
*Substance use*							
Never used ≥ 50 cigarettes ^x^	3,169	(80.0 %)	65	(24.2 %)	12.56	(9.40–16.78)	<.001
Lifetime use of ≥ 50 cigarettes	792	(20.0 %)	204	(75.8 %)			
Age of first cigarette smoking, *M (SD)* ^y^	15.1	(2.4)	14.7	(2.9)	0.94	(0.90–0.99)	.026
No use of cigarettes (previous 12 months) (Ref) ^z^	2,774	(70.0 %)	50	(18.6 %)			
Occasional (non-daily) cigarette use	1,187	(30.0 %)	219	(81.4 %)	10.24	(7.47–14.02)	<.001
No use of tobacco product other than cigarettes (previous 12 months) (Ref) ^aa^	2,189	(55.3 %)	68	(25.3 %)			
Use of tobacco product other than cigarettes	1,772	(44.7 %)	201	(74.7 %)	3.65	(2.75–4.84)	<.001
Never used ≥ 12 alcoholic drinks ^ab^	421	(11.1 %)	13	(4.9 %)			
Lifetime use of ≥ 12 alcoholic drinks	3,366	(88.9 %)	250	(95.1 %)	2.35	(1.33–4.13)	.003
Age of first drink, *M (SD)* ^ac^	14.6	(1.9)	14.0	(2.03)	0.87	(0.83–0.92)	<.001
Alcohol use—no use or not at-risk (AUDIT-C) (previous 12 months) (Ref) ^ad^	1,374	(35.0 %)	58	(21.8 %)			
Alcohol use—at-risk	2,550	(65.0 %)	208	(78.2 %)	1.93	(1.43–2.60)	<.001
Never used cannabis ^ae^	1,433	(36.2 %)	194	(72.1 %)			
Lifetime use of cannabis	2,522	(63.8 %)	75	(27.9 %)	4.55	(3.46–5.99)	<.001
Age of first cannabis use, *M (SD)* ^af^	16.2	(1.8)	15.6	(2.1)	0.84	(0.77–0.90)	<.001
No cannabis use (previous 12 months) (Ref) ^ag^	3,125	(18.9 %)	119	(44.2 %)			
No problem use (CUDIT)	701	(17.7 %)	95	(35.3 %)	3.56	(2.69–4.72)	<.001
Problem use (CUDIT)	134	(3.4 %)	55	(20.4 %)	10.78	(7.50–15.50)	<.001
Never used illicit drugs other than cannabis (Ref) ^ah^	3,517	(89.7 %)	181	(68.0 %)			
Lifetime use of illicit drugs other than cannabis	406	(10.3 %)	85	(32.0 %)	1.07	(3.08–5.37)	<.001
*Personality*							
No attention deficit syndrome (ASRS) (Ref) ^ai^	3,820	(96.6 %)	257	(95.9 %)			
Attention deficit syndrome	135	(3.4 %)	11	(4.1 %)	1.21	(0.65–2.27)	.550
Sensation seeking (BSSS total score, range 1–5), *M (SD)* ^aj^	3.0	(0.8)	3.3	(0.9)	1.56	(1.34–1.82)	<.001
Aggression (ZKPQ, subscale, range 0–10), *M (SD)* ^ak^	4.0	(2.2)	4.6	(2.1)	1.13	(1.07–1.20)	<.001
Sociability (ZKPQ, subscale, range 0–10), *M (SD)* ^al^	5.7	(2.3)	6.4	(1.9)	1.15	(1.09–1.22)	<.001
Anxiety (ZKPQ, subscale, range 0–10), *M (SD)* ^am^	1.9	(1.9)	2.0	(2.0)	1.02	(0.95–1.08)	.636
Peer pressure (PPI total score, range −3–+3), *M (SD)* ^an^	0.3	(0.4)	0.4	(0.4)	1.46	(1.06–2.00)	.020
No anti-social personality disorder (Ref) ^ao^	3,429	(87.6 %)	196	(74.5 %)			
Anti-social personality disorder	484	(12.4 %)	67	(25.5 %)	2.42	(1.81–3.25)	<.001

Separate binary logistic regression model for each baseline variable. Values are n (%) unless stated otherwise. Missing values: ^a^ *n* = 1, ^b^ *n* = 74, ^c^ *n* = 28, ^d^ *n* = 31, ^e^ *n* = 30, ^f^ *n* = 134, ^g^ *n* = 62, ^h^ *n* = 63, ^i^ *n* = 29, ^j^ *n* = 29, ^k^ *n* = 42, ^l^ *n* = 308, ^m^ *n* = 49, ^n^ *n* = 55, ^o^ *n* = 65, ^p^ *n* = 72, ^q^ *n* = 31, ^r^ *n* = 12, ^s^ *n* = 15, ^t^ *n* = 16, ^u^ *n* = 23, ^v^ *n* = 24, ^w^ *n* = 60, ^x^ *n* = 0, ^y^ *n* = 2028, ^z^ *n* = 0, ^aa^ *n* = 0, ^ab^ *n* = 6, ^ac^ *n* = 175, ^ad^ *n* = 40, ^ae^ *n* = 6, ^af^ *n* = 2,605, ^ag^ *n* = 1, ^ah^ *n* = 41, ^ai^ *n* = 7, ^aj^ *n* = 3, ^ak^ *n* = 5, ^al^ *n* = 5, ^am^ *n* = 5, ^an^ *n* = 81, ^ao^ *n* = 54

*ASRS* Item Screener of the Attention Deficit Syndrome Self Report Scale, *AUDIT-C* Alcohol Use Disorders Identification Test–Consumption, *BSSS* Brief Sensation Seeking Scale, *CI* confidence interval, *CUDIT* Cannabis Use Disorder Identification Test, *IPAQ* International Physical Activity Questionnaire, *M* mean, *MDI* Major Depressive Inventory, *OR* Odds Ratio, *PPI* Peer Pressure Inventory, *Ref* reference category, *SD* standard deviation, *SF-12* Short-Form Health Survey, *ZKPQ* Zuckerman-Kuhlman Personality Questionnaire

### Predictors of the onset of daily tobacco use

Between baseline and follow-up, 269 (6.4 %) participants progressed to daily cigarette smoking. Table 1 shows the results of the separate logistic regression analyses indicating individual associations between predictors and the onset of cigarette use. Table 2 presents the category-specific models and the final model. According to the final model, having used cannabis and/or having occasionally smoked cigarettes during the 12 months before baseline, and a lifetime use of more than 50 cigarettes were associated with a progression to daily cigarette use. Beyond substance use, the following variables predicted a higher probability of progression to daily cigarette use: a lower educational level, having no religious affiliation (as opposed to Christian religion), having grown up with only one parent and a step-parent (as opposed to both parents), having had peers with psychiatric problems, and a higher sociability. All the variables included in the final model explain 27.9 % of the variance. The group of variables related to substance use represented the strongest predictors, with a variance explanation of 25.1 %.

**Table 2:** Multivariate associations between baseline variables and the onset of daily cigarette use

Variable categories and variables	Category-specific models		Overall model	
	*OR (95 % CI)*	*p*	*OR (95 % CI)*	*p*
*Socio-demographics*				
Lower educational level (Ref)				
Higher educational level	0.51 (0.37–0.71)	<.001	0.45 (0.32–0.65)	<.001
Living with parent or parents (Ref)				
Living alone	1.45 (0.72–2.92)	.294		
Living with partner	1.69 (0.84–3.41)	.144		
Living with friends or in institution	2.25 (1.32–3.84)	.003		
*Religion and spirituality*				
Christian religion (Ref)				
Muslim religion	1.40 (0.71–2.73)	.332	1.53 (0.74–3.17)	.250
Other religion	1.26 (0.54–2.92)	.579	1.01 (0.40–2.56)	.982
No religion	1.48 (1.09–2.01)	.013	1.42 (1.03–1.97)	.035
Atheist (Ref)				
Agnostic	0.85 (0.58–1.25)	.410		
Unsure what to think about god	0.995 (0.66–1.50)	.983		
Believe in god but not practicing	0.86 (0.61–1.21)	.393		
Believe in god and practicing	0.49 (0.29–0.84)	.010		
*Health and health behaviour*				
Depression (MDI, scale 0–50)	1.03 (1.01–1.04)	.001		
*Social context*				
Grew up with both parents (Ref)				
…with parent and step-parent	2.60 (1.71–3.96)	<.001	2.16 (1.34–3.49)	.002
…with one parent	1.57 (1.12–2.20)	.008	1.27 (0.87–1.85)	.214
…with adoptive or foster parents or in institution	0.95 (0.29–3.08)	.928	0.71 (0.21–2.43)	.583
No psychiatric problem in peer/s at age of 15 (Ref)				
Psychiatric problem in peer/s at age of 15	1.97 (1.53–2.54)	<.001	1.35 (1.02–1.78)	.038
*Substance use*				
Never used ≥ 50 cigarettes				
Lifetime use of ≥ 50 cigarettes	4.88 (3.37–7.08)	<.001	4.22 (2.89–6.16)	<.001
No use of cigarettes during previous 12 months				
Occasional (non-daily) cigarette use	3.02 (2.01–4.54)	<.001	3.02 (1.99–4.58)	<.001
No cannabis use (previous 12 months) (Ref)				
No problem use (CUDIT)	1.41 (1.03–1.92)	.031	1.52 (1.10–2.09)	.011
Problem use (CUDIT)	3.00 (2.02–4.47)	<.001	3.06 (2.00–4.67)	<.001
*Personality*				
Sensation seeking (BSSS total score, range 1–5), *M (SD)*	1.36 (1.15–1.60)	<.001		
Aggression (ZKPQ, subscale, range 0–10)	1.07 (1.01–1.14)	.025		
Sociability (ZKPQ, subscale, range 0–10)	1.12 (1.06–1.19)	<.001	1.12 (1.04–1.20)	.002
No anti-social personality disorder (Ref)				
Anti-social personality disorder	1.84 (1.33–2.52)	<.001		

Nagelkerke’s *R* ^*2*^: overall model: 0.279, demographics: 0.018, religion and spirituality: 0.012, health and health behaviour: 0.006, social context: 0.032, substance use: 0.251, personality: 0.046

*BSSS* Brief Sensation Seeking Scale, *CI* confidence interval, *CUDIT* Cannabis Use Disorder Identification Test, *MDI* Major Depressive Inventory, *OR* Odds Ratio, *Ref* reference category, *ZKPQ* Zuckerman-Kuhlman Personality Questionnaire

## Discussion

This study aimed to examine the role of cannabis use in the progression of tobacco use, as previously investigated in several studies dedicated to the reverse gateway hypothesis [3–5, 47]. Compared to previous studies, we accounted for a broad range of predictor variables, including variables that have not been previously studied in this context, e.g., religiosity and several personality dimensions. With regard to the onset of daily cigarette smoking, cannabis use, i.e., more pronounced cannabis use disorder symptoms according to the CUDIT, remained among the strongest predictors aside from the lifetime use of more than 50 cigarettes and occasional cigarette use. This strong association between cannabis and tobacco use may be partially explained by the fact that more than 90 % of the Swiss cannabis users smoke it mixed with tobacco [15]. Thus, in line with previous qualitative [10] and quantitative findings [7], our results suggest that the way of administration of cannabis and tobacco, particularly smoking cannabis joints mixed with tobacco, plays an important role in young adults’ onset of daily cigarette use, making these findings particularly relevant for countries in which cannabis is mainly co-administered with tobacco. Moreover, we found that the use of tobacco products other than cigarettes (i.e., water pipes (shisha, smoked only with tobacco), snus, snuff, chewing tobacco, cigars/cigarillos, and tobacco pipes) played a less important role than cannabis use for the onset of daily cigarette use. This is in line with the study of Agrawal and Lynskey [7] in which smoking tobacco was significantly associated with cannabis use and dependence whereas the use of smokeless tobacco was not.

In our study, the factors referring to a genetic vulnerability such as a psychiatric disorder of the father/mother or externalizing and/or delinquent personality traits (anti-social personality, aggression, sensation seeking, and attention deficit syndrome) did not remain significant in the overall predictor model. This is in line with the conclusion of Ramo and colleagues [48] who found in their systematic literature review that not genetic but environmental factors appear to account for the largest variance in the co-use of tobacco and cannabis. Although we did not assess peers’ substance use and delinquency as an explaining factor [19], in our models for the onset of daily cigarette smoking the psychiatric problems of peers at age 15 were still of higher relevance than genetic factors. Similarly, to grow up with one parent and step-parent remained as a further context factor in the overall model. More specific socio-economic factors or factors indicating a change of these [21] during adolescence did not enter the overall model.

Among the religion and spirituality factors, having no religion was a relevant predictor for the onset of daily cigarette smoking. Whether substantial gains or losses in religiosity from childhood to adulthood occurred, a predictor that has been reported to be associated with substance use and misuse in the general U.S. population [20], was not assessed in our study. In line with Agrawal et al. [11], to believe in God and to practice religion was the other significant predictor in the category-specific religion and spirituality model, but this variable did not enter the overall predictor model.

Considering the amount of variance explained by the category-specific models, substance abuse explained the major part of variance (25.1 %) compared to the other categories (0.6 % to 4.6 %).

One limitation of this study is that the participants were only observed for a relatively short time period because they were reassessed at one time point after 15 months. In addition, there was a period of approximately three months that was not included in the assessments because the follow-up assessment only examined the preceding 12 months. In order to analyse potential moderators and mediators in the reverse gateway scenario, cohort studies including multiple assessments over a longer time period are required. A further limitation is the dichotomized outcome variable (daily cigarette smoking vs. non-daily cigarette smoking). This implicates that a group of 216 smokers who smoked cigarettes on 5 to 6 days a week already at baseline were treated as non-daily smokers and therefore includes in the analysis. These occasional smokers may have had a particularly high chance to proceed to daily cigarette smoking and to be treated as participants with “onset of daily cigarette smoking” which may seem an artificial transition, given they already smoked already nearly daily at baseline.

## Conclusions

According to our findings, appropriate interventions to prevent young adults from daily cigarette use should specifically address occasional cigarette smokers and users of cannabis that mix and smoke these substances together.

## Abbreviations

ASRS: Item Screener of the Attention Deficit Syndrome Self Report Scale
AUDIT-C: Alcohol Use Disorders Identification Test–Consumption
BSSS: Brief Sensation Seeking Scale
C-SURF: Cohort Study on Substance Use Risk Factors
CUDIT: Cannabis Use Disorder Identification Test
IPAQ: International Physical Activity Questionnaire
MDI: Major Depressive Inventory
PPI: Peer Pressure Inventory
Ref: reference category
SF-12: Short-Form Health Survey
ZKPQ: Zuckerman-Kuhlman Personality Questionnaire
